# Preparation of FeCo/C-N and FeNi/C-N Nanocomposites from Acrylamide Co-Crystallizates and Their Use as Lubricant Additives

**DOI:** 10.3390/mi13111984

**Published:** 2022-11-16

**Authors:** Igor E. Uflyand, Victoria E. Burlakova, Ekaterina G. Drogan, Igor Yu. Zabiyaka, Kamila A. Kydralieva, Gulsara D. Kugabaeva, Gulzhian I. Dzhardimalieva

**Affiliations:** 1 Department of Chemistry, Southern Federal University, 344006 Rostov-on-Don, Russia; 2 Department of Chemistry, Don State Technical University, 344010 Rostov-on-Don, Russia; 3 Moscow Aviation Institute, National Research University, 125993 Moscow, Russia; 4 Institute of Problems of Chemical Physics, Russian Academy of Sciences, Chernogolovka, 142432 Moscow, Russia

**Keywords:** FeCo-polyacrylamide complex, FeNi-polyacrylamide complex, frontal polymerization, FeCo/C-N nanoparticles, FeNi/C-N nanoparticles, thermolysis, N-doped carbonized shell

## Abstract

FeCo and FeNi nanoalloy particles encapsulated in a nitrogen-doped carbonized shell (FeCo/C-N and FeNi/C-N) were synthesized by thermolysis at 400 °C of polyacrylamide complexes after frontal polymerization of co-crystallizate of Fe and Co or Ni nitrates and acrylamide. During the thermolysis of polyacrylamide complexes in a self-generated atmosphere, Co(II) or Ni(II) and Fe(III) cations are reduced to form FeCo and FeNi nanoalloy particles, while polyacrylamide simultaneously forms a nitrogen-doped carbon shell layer. This unique architecture provides high chemical and thermal stability of the resulting nanocomposites. The average crystallite size of FeCo and FeNi nanoparticles is 10 and 12 nm, respectively. The nanocomposites were studied by X-ray diffraction, atomic force microscopy, scanning electron microscopy, and high-resolution transmission electron microscopy. The nanocomposites have been tested as antifriction and antiwear additives in lubricating oils. The optimal concentrations of nanoparticles were determined, at which the antifriction and antiwear properties of the lubricant manifest themselves in the best possible way.

## 1. Introduction

Transition metal nanoparticles (NPs) encapsulated in nitrogen-doped carbon (C-N), obtained from precursors containing nitrogen, carbon, and earth-abundant transition metals (in particular, Co, Fe, Ni, and Mn), are of wide interest due to their natural abundance and cheapness [1,2,3,4,5,6]. These materials are usually obtained by thermolysis of metal-organic complexes [3,4,7], metal salts and carbon materials [5,8], and metal-organic frameworks [9,10,11,12]. However, such uncontrolled thermolysis will inevitably lead to excessive encapsulation or exposure of NPs. In addition, exposed NPs are easily inactivated during the reaction, resulting in poor stability. Therefore, there is an urgent need to develop a technology that can effectively encapsulate NPs with nitrogen-doped carbon. Previous studies [13] showed that one of the effective methods for the synthesis of metal nanocomposites is the thermal transformation of metal-containing monomers through an intermediate stage of monomer polymerization in the solid phase. During the reaction, the formation of metal NPs and their stabilizing polymer matrix are combined.

Bimetallic nanostructures in a nitrogen-doped carbon matrix represent a new class of polyfunctional materials with high magnetic, catalytic, tribological, damping, and other properties [14,15,16]. As an example, the most typical examples of bimetallic nanocomposites used as antifriction materials are presented in Table 1. 

Although NPs of metal alloys are considered promising materials for various applications, they are sensitive to the atmosphere due to a very large surface-to-volume ratio, i.e., their surfaces are easily oxidized. NPs encapsulated in carbon materials are highly resistant to environmental influences since their graphite shell protects NPs from degradation in the atmosphere [31,32].

One of the most promising methods for obtaining NPs is the formation of NPs in situ at the stage of condensation of a polymer–inorganic matrix by frontal polymerization (FP) [33,34]. This approach made it possible to solve the problem of introducing NPs of sufficiently high density into the polymer matrix [35]. FP for monomeric complexes M(NO_3_)_2_·4AAm·2H_2_O (M = Co(II), Ni(II), Fe(III), and AAm is acrylamide) was first studied in 2004 by Dzhardimalieva et al. [36]. As shown by the example of the acrylamide complex of Co(II) nitrate, AAm complexes of metal nitrates are convenient objects of FP in the structural and chemical aspect: the orientation of their molecules is optimal for the formation of chemical bonds between them, and the growth of chains occurs in the plane of peculiar “blanks”—stacks, which are densely packed molecules of metal monomers parallel to each other.

In this work, to obtain bimetallic (FeCo and FeNi) nanocomposites, we used a two-stage approach through FP of FeCoAAm and FeNiAAm co-crystallizates followed by thermolysis. This approach consists in combining the synthesis of bimetallic NPs and a nitrogen-doped carbon shell, which stabilizes them in situ during FP of metal-containing monomers—acrylamide co-crystallizates in the solid phase, and subsequently controlled thermolysis of the formed metallopolymers. The resulting nanocomposites were studied by powder X-ray diffraction, atomic force microscopy, scanning electron microscopy, and high-resolution transmission electron microscopy to determine their chemical structure and morphology. In addition, nanocomposites have found applications as antifriction and antiwear additives to lubricants.

## 2. Materials and Methods

### 2.1. Materials

Fe(NO_3_)_3_∙9H_2_O (≥98%), Co(NO_3_)_2_∙6H_2_O (98%), and acrylamide (AAm) (≥98%) were purchased from Sigma-Aldrich Chemie GmbH, Steinheim, Germany and were used without additional purification. Benzene (chemically pure, Chimmed, Moscow, Russia) and diethyl ether (chemically pure, Chimmed, Moscow, Russia) were purified and distilled according to the standard procedure.

### 2.2. Material Synthesis 

#### 2.2.1. Synthesis of Monomer Fe(III)/Co(II) and Fe(III)/Ni(II) Complexes 

Acrylamide complexes of metal nitrates M(NO_3_)_n_∙4AAm∙2H_2_O, where M = Co(II), Ni(II), Fe(III), served as precursors for the synthesis of the co-crystallizate system. Co-crystallization of FeCoAAm and FeNiAAm was carried by thoroughly mixing Fe(NO_3_)_3_∙9H_2_O (2.50 g, 6.19 × 10^−3^ mol) and Co(NO_3_)_2_∙6H_2_O (1.80 g, 6.19 × 10^−3^ mol) or Ni(NO_3_)_2_∙6H_2_O (1.80 g, 6.19 × 10^−3^ mol) in the presence of CH_2_=CHCONH_2_ (AAm) (2.197 g, 3.1 × 10^−3^ mol) at a molar ratio of Fe(III) and Co(II) or Ni(II) nitrates/AAm equal to 1:1:5 according to the procedure as described earlier [37].

#### 2.2.2. Preparation of the Polyacrylamide Complexes 

FP of monomeric co-crystallized FeCoAAm or FeNiAAm metal complexes was carried out at atmospheric pressure in a self-generated atmosphere by short-term thermal perturbation (Wood’s melt) in the end part of the billet. Thermal initiation (437 K) for 10–12 s led to the appearance of a mobile polymerization front (453 K). The reaction was monitored visually by the movement of the staining boundary along the sample. At the output, polymerized reaction products were obtained in the form of cylindrical close-packed powder blanks (FeCoPolyAAm or FeNiPolyAAm).

#### 2.2.3. Preparation of FeCo/C-N and FeNi/C-N Nanocomposites 

A sample of the polyacrylamide complex was dried in a desiccator at 25 °C in the presence of P_2_O_5_ as a dehydrating agent, while the by-products of the reaction H_2_O and H_3_PO_4_ were continuously removed. Next, the dried product was subjected to thermolysis at 400 °C in a nitrogen atmosphere. Thermolysis was carried out with volumetric heating in an oven for 150 min. At the output, a highly dispersed powdered nanocomposite (FeCo/C-N or FeNi/C-N) was obtained. 

### 2.3. Characterization

A CHNOS Vario EL cubic analyzer (Elementar Analysensysteme GmbH, Langenselbold, Germany) was used for elemental analysis. Metals were determined on AAS3 equipment (VEB Feinmesszeug Fabrik, Zeiss, Germany). The study was carried out by atomic absorption spectral analysis using acetylene-air flame spraying for determination. 

FTIR spectroscopic analysis was performed using a Bruker Tensor 37 FTIR spectrometer (Bruker Corporation, Billerica, MA, USA) with a resolution of 2 cm^−1^ and Softspectra data analysis software (Bruker Corporation, Billerica, MA, USA). Thermogravimetry (TG) and differential scanning calorimetry were performed on a PerkinElmer Diamond derivatograph (PerkinElmer, Waltham, MA, USA) in air at atmospheric pressure with an α-Al_2_O_3_ standard at a heating rate of 10°/min in the range of 20–800 °C.

X-ray diffraction (XRD) analysis was performed on a Thermo Fisher Scientific ARL X’TRA powder diffractometer (Thermo Fisher Scientific Inc., Waltham, MA, USA) using CuKα radiation (λ_Cu_ = 1.54184 Å) in the angle range 2*θ* = 5–80° at a scanning speed of 5 deg/min, and a temperature of 300 K was used to determine the phase composition and sizes of crystallites. The crystallite size (*D*, nm) was determined by the Debye-Scherrer Equation (1):

(1) $$D=\frac{k\lambda }{\beta \cos\theta }$$

where *k* is a dimensionless coefficient (Scherer’s constant ~0.9); *β* is the peak width at half maximum intensity; *λ* is the wavelength of X-ray radiation; *θ* is the diffraction angle.

The interplanar spacing (*d*, nm) between atoms was determined using the Wolf–Bragg Equation (2):

(2) $$d\;=\;\frac{\lambda }{2\;\cdot \sin\theta }$$

Atomic force microscopy (AFM, PHYWE Systeme GmbH & Co. KG, Göttingen, Germany) was performed on a PHYWE Compact scanning probe microscope in the semi-contact mode with an aluminum-coated single-crystal silicon probe. The tests were carried out at a resonant frequency of 190 ± 60 kHz, a constant hardness of 48 N/m, and a scan rate of 0.3 ms/line. Sample preparation included ultrasonic treatment of metal powders in ethanol for 30 min, followed by the application of a colloidal solution to a coverslip and drying in air.

Scanning electron microscopy (SEM) was examined using a Zeiss CrossBeam 340 high-resolution dual-beam scanning electron microscope (Carl Zeiss AG, Jena, Germany) with a Schottky electron emission source. Energy-dispersive X-ray spectroscopy microanalysis (EDX) was performed on an Oxford X-max 80 microanalyzer (Oxford Instruments, Abingdon, Oxfordshire, UK) with an electron probe energy of ≤10 keV.

The microstructure of the nanocomposites and their elemental composition were studied by transmission electron microscopy (TEM). TEM analysis was performed on a Bruker Nano GmbH (Bruker Corporation, Billerica, MA, USA). The samples were prepared as follows: a suspension of the powder in hexane was prepared, deposited on a carbon-coated copper grid, and the solvent was dried in air.

### 2.4. Preparation of the Lubricant Composition

Lubricant compositions were obtained by suspending additives in liquid paraffin (LP), which were used in small amounts at the sliding boundary of a steel-to-steel friction pair in accordance with ASTM G-99. LP was used as the basis of the lubricant composition since it has no impurities and low tribotechnical characteristics. The weighing of LP and additives was carried out on an analytical balance. The additives were dispersed in LP using a PSB-Hals ultrasonic shaker for 15 min. The percentage of the additive in LP was 0.05, 0.1, 0.2, and 0.5 wt.%.

### 2.5. Tribological Tests

The antiwear characteristics of a steel-steel friction pair in a lubricant composition based on LP and additives were studied on a four-ball friction machine (FBFM, LLC Neftekhimmashsystemy, Ryazan, Russia). The steel-steel friction pair at FBFM was a point contact of balls with a diameter of 0.53″ (12.7 mm) made of ShKh-15 steel according to GOST 801-78, heat-treated to a hardness of HRC 62-66. All parts of the machine that came into contact with the lubricant during the test were washed with solvent and air-dried before starting the next test. The three bottom balls were fixed in a bowl filled with lubricant. The upper ball was fixed in the machine spindle. The anti-wear properties of the lubricant composition were determined by the wear scar diameter (WSD) of each of the three balls using a Carl Zeiss AxioVert.A1 optical microscope (Carl Zeiss AG, Jena, Germany). Tests on FBFM were carried out for 3600 s at a constant load of 20 N/m.

A pin-on-disk testing machine of the UMT-200 type (Research and Production Center “Konvers-resurs”, Moscow, Russia) was used to study the antifriction properties of the lubricant composition. The speed of rotation of the disk without load does not exceed 2900 rpm with a pressing force of 0 to 200 kg. The friction unit is a steel disk with a diameter of 50 mm and three steel pins with a diameter of 8 mm. In the case of thick lubricant, a pin with a diameter of 20 mm was used. Before each test, the surfaces of the disk and pins were ground and polished with 600 grit sandpaper, then washed with distilled water, cleaned with hexane, and dried at room temperature in air. Each test was repeated three times. 

## 3. Results and Discussion 

### 3.1. Preparation and Frontal Polymerization of Acrylamide Co-Crystallizates

**Figure 1:**
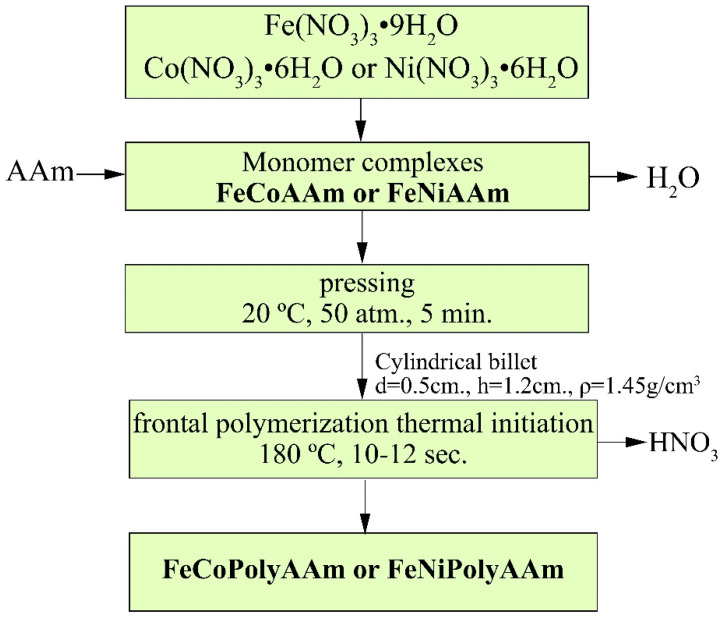
Scheme for the synthesis of monomeric complexes and their frontal polymerization.

In the present work, acrylamide co-crystallizates were obtained by thoroughly mixing Fe(NO_3_)_3_∙9H_2_O and Co(NO_3_)_2_∙6H_2_O or Ni(NO_3_)_2_∙6H_2_O in the presence of AAm and used as monomers for FP according to Figure 1.

**Figure 2:**
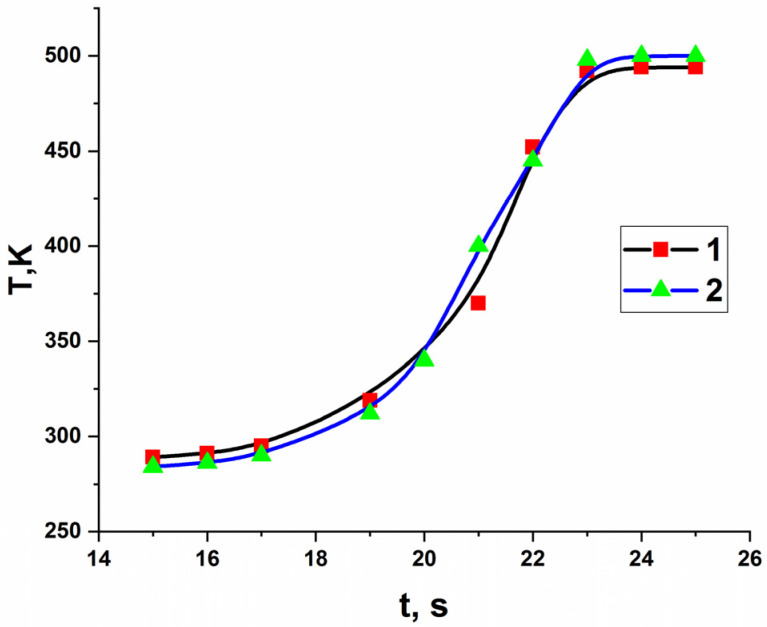
Temperature profile of the polymerization process in the frontal mode for FeCoAAm (1) and FeNiAAm (2) (T = 437 K, d = 1.2 cm, r = 1.38 g/cm^3^).

During FP, acrylamide co-crystallizates are converted into polymer complexes in a localized reaction zone and a layer-by-layer regime, spreading throughout the entire volume. Simultaneously, the precursors and their stabilizing carbon shells were synthesized and fused in situ based on the copolymerization reaction of a metal-containing monomer in the solid phase. It was found that a change in the initiation temperature of acrylamide co-crystallizates does not significantly affect the front propagation velocity (Figure 2), which is consistent with the data for the Co(II) acrylamide complex [36].

A detailed study of the mechanism of polymerization of the monomeric complex CoAAm showed [38] that this process is preceded by a phase transition—melting of the metal-containing monomer. In this case, the combination of endo- and exothermic processes occurring near the reaction front leads to gross exothermic effects, which ultimately maintain the frontal regime of chemical transformation.

Qualitative phase analysis by time-resolved XRD analysis in the polymerization wave mode showed that up to 437 K, there are no changes in the diffraction pattern of the initial FeCoAAm crystalline monomer. The weak reflections appearing above 437 K in the diffraction pattern of FeCoAAm indicate the nucleation of a new phase. When the sample is kept at a constant temperature of 437 K, an increase in the intensity of the lines of this phase is observed. Then an additional row of peaks belonging to another high-temperature phase appears in the diffraction pattern. At 453 K, no transformations of the peaks are observed, and a pure high-temperature anhydrous phase appears (due to the elimination of water molecules according to [36]), which precedes polymerization. The final X-ray pattern corresponds to an amorphous polymer; at the same time, it has a well-defined peak with an interplanar distance of 8.9 Å, which is consistent with the peak in the monomer and indicates some crystallinity of the resulting product. According to the phase analysis of the process of FP of the bimetallic complex, three main stages of chemical transformations are assumed to occur (Scheme 1). 

I. Monomer dehydration: 

[Fe(CH_2_=CHC(O)NH_2_)_4_∙(H_2_O)_3_∙(NO_3_)_3_]∙[Co(CH_2_=CHC(O)NH_2_)_4_∙(H_2_O)_2_∙(NO_3_)_2_](solid) → [Fe(CH_2_=CHC(O)NH_2_)_4_∙(NO_3_)_3_]∙[Co(CH_2_=CHC(O)NH_2_)_4_∙(NO_3_)_2_](solid) + 5(H_2_O)(liquid)

II. Polymerization of the dehydrated complex (437 K). Interaction of H_2_O vapors with the NO_3_ anion and NH_2_ group of the AAm ligand, leading to the formation of NH_3_ and HNO_3_ vapors:

n{[Fe(CH_2_=CHC(O)NH_2_)_4_∙(NO_3_)_3_]∙[Co(CH_2_=CHC(O)NH_2_)_4_∙(NO_3_)_2_]} → –H_2_O(vapor) → {[Fe(CH_2_=CHC(O)NH_2_)_4_]∙[Co(CH_2_=CHC(O)NH_2_)_4_]}_m_ + 2NH_3_(vapor) + HNO_3_(vapor)

III. Thermal-oxidative destruction of the resulting polymer (≥453 K) due to interaction with HNO_3_ vapor and reactive products of its decomposition.

Scheme 1. Main stages of thermal transformations of FeCoAAm complex.

**Figure 3:**
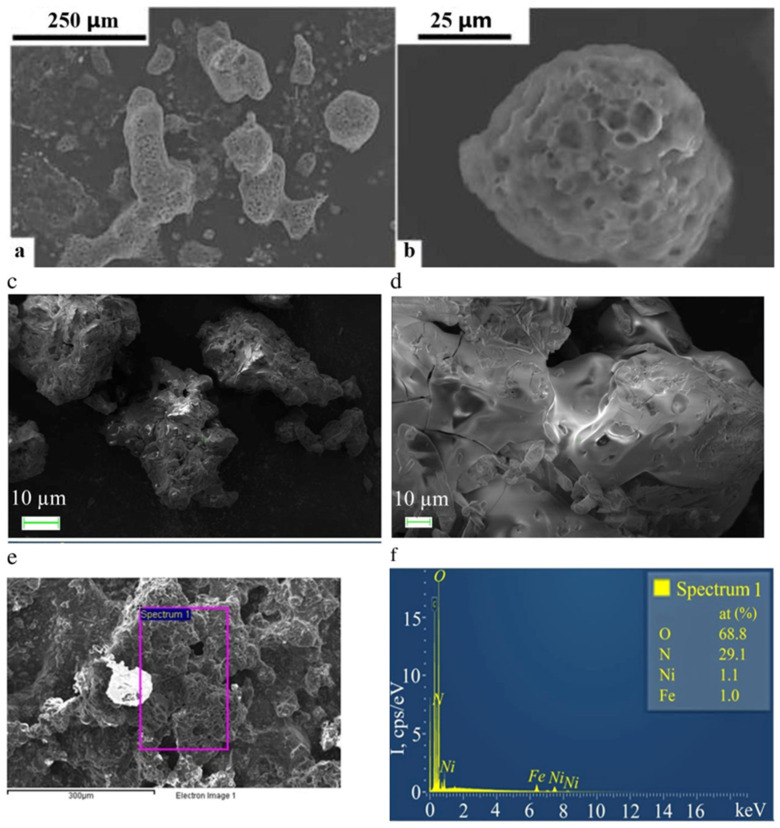
SEM micrographs of FeCoPolyAAm (**a**,**b**) and FeNiPolyAAm (**c**,**d**) samples and EDX data of FeNiPolyAAm sample (**e**,**f**).

SEM images of the FeCoPolyAAm and FeNiPolyAAm complexes (Figure 3) demonstrate the formation of ellipsoid-type porous bulk conglomerates with an average size of 100 µm. EDX measurements revealed that the composition of the complexes is in accordance with the precursor’s molecular ratio (1/1) used in the synthesis procedure.

**Figure 4:**
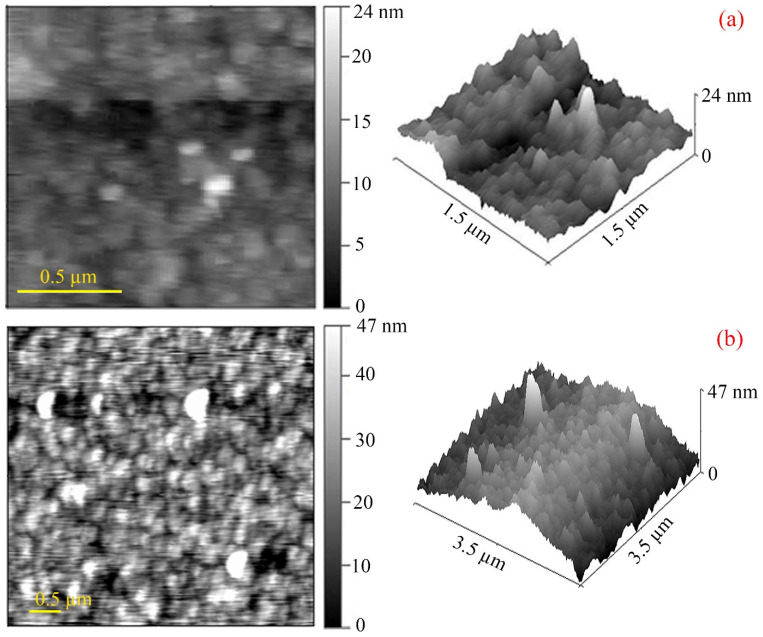
AFM image of FeCoPolyAAm (**a**) and FeNiPolyAAm (**b**) samples.

AFM visualization of the studied FeCoPolyAAm and FeNiPolyAAm powders indicates their nanoscale parameters (Figure 4). The particle surface has a low roughness; FeCoPolyAAm powder particles have a size of 100 to 200 nm with a maximum height of pyramidal particles of about 24 nm. The results of AFM of FeNiPolyAAm powders showed the presence of elongated cone-shaped pointed particles with a maximum size of up to 47 nm, located close to each other.

### 3.2. Tribological Testing of Polymer Complexes

**Figure 5:**
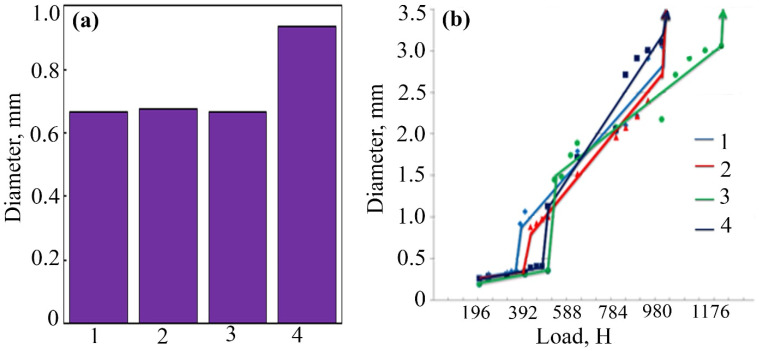
The results of the study of anti-wear properties (**a**), bearing and maximum load capacity (**b**) of LP with the addition of FeCoPolyAAm: 1—LP, 2—LP + 0.025% FeCoPolyAAm, 3—LP + 0.05% FeCoPolyAAm, 4—LP + 0.1% FeCoPolyAAm.

**Figure 6:**
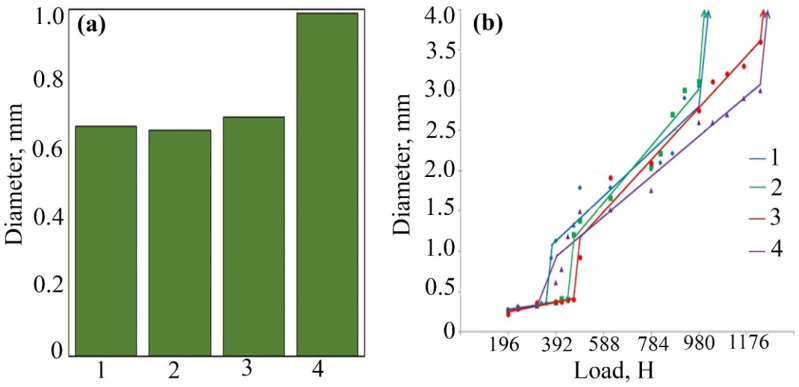
The results of the study of anti-wear properties (**a**), bearing and maximum load capacity (**b**) of LP with the addition of FeNiPolyAAm: 1—LP, 2—LP + 0.025% FeNiPolyAAm, 3—LP + 0.05% FeNiPolyAAm, 4—LP + 0.1% FeNiPolyAAm.

The use of FeCoPolyAAm and FeNiPolyAAm as antifriction additives shows that during the hourly tribological tests, WSD dependences on concentration (Figure 5a and Figure 6a) were obtained. The addition of the FeCoPolyAAm additive increased WSD at all studied concentrations from 0.6402 to 0.9954 mm (Figure 5a). However, when modifying the base LP by adding 0.025% FeCoPolyAAm, it was possible to increase the critical load by 11.05% from 353 to 392 N, which indicates an improvement in the tribological properties of the material, but the welding load does not change and remains at the level of 980 N (Figure 5b). With an increase in the FeCoPolyAAm concentration to 0.05%, a significant improvement in the tribological properties of the lubricant is noted; for example, the critical load increases by 38.81% from 353 to 490 N, and the welding load increases by 26.02% compared with pure LP from 980 to 1235 H (Figure 5b). At the studied loads, WSDs up to the critical load was somewhat smaller than those of pure LP and LP with additives. After the critical load, a sharp jump in WSDs was observed; however, closer to the welding load (after 784 N), WSD becomes smaller than for other studied concentrations and pure LP. After modification of the pure LP with 0.1% FeCoPolyAAm, an improvement in the tribological properties of the lubricant compared with pure LP was revealed, so the critical load increased by 31.73% from 353 to 465 N. Compared with the composition containing 0.05% additive, the load welding decreases by 20.65% from 1235 to 980 N. After a critical load, a sharp jump in the IRR was observed; however, closer to the welding load (after 784 N), the WSD becomes smaller than for the other concentrations tested and pure LP. WSDs before the critical load are generally almost the same as for a concentration of 0.05%, but after the critical load they are basically worse than for all other samples. Thus, the introduction of a small amount of the FeCoPolyAAm additive made it possible to significantly improve the tribological properties of the base LP.

The addition of FeNiPolyAAm in an amount of 0.025% increases the critical load and, at the same time, reduces the wear of steel balls; however, after overcoming the load of 784 N, the data on the graph became equal to those of pure LP (Figure 6b). Doubling the concentration to 0.05% gave more satisfactory results. The welding load and critical load increase significantly compared with other tested concentrations and pure LP: the welding load is 1039 N, and the critical load is 617 N. A subsequent increase in concentration to 0.1% also increases the critical load compared with pure LP by 25%. A significant increase in welding load and a reduction in ball wear were noted, in contrast to other concentrations tested (Figure 6b).

**Figure 7:**
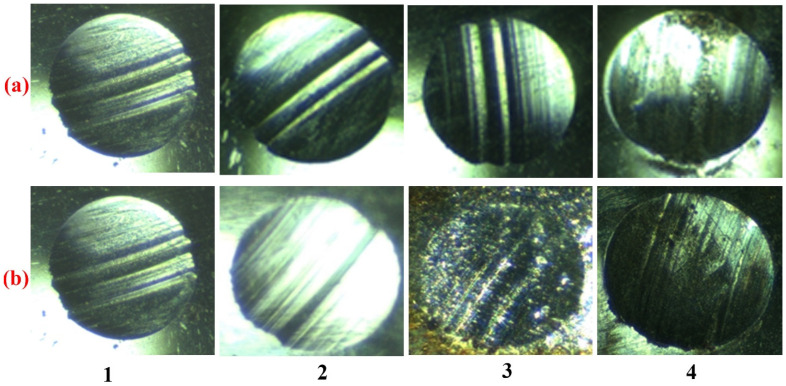
The results of optical microscopy of steel balls after 1 h of testing for FBFM in LP with additions of FeCoPolyAAm (**a**) and FeNiPolyAAm (**b**) with concentrations: 1—LP, 2—0.025%, 3—0.05%, 4—0.1%.

The photographs taken with an optical microscope (Figure 7) show that without additives, as well as with concentrations of 0.025% and 0.05% FeCoPolyAAm and FeNiPolyAAm, a deep groove is observed on the balls, indicating large wear of the friction surface. With an increase in the concentration of the additive to 0.1%, some plowing of the surface is noted with some fragments of corrosion damage but with less wear (Figure 7).

**Figure 8:**
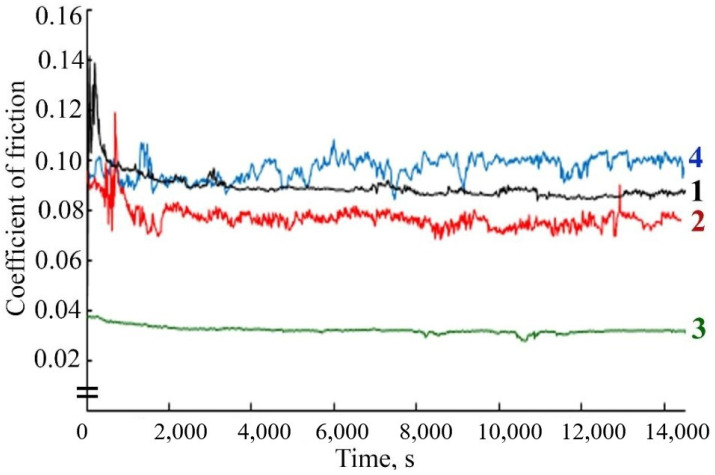
Change in friction coefficient over time when using the lubricant: 1—LP, 2—LP + 0.025% FeCoPolyAAm, 3—LP + 0.05% FeCoPolyAAm, 4—LP + 0.1% FeCoPolyAAm.

As follows from the results of the study of the tribological behavior of FeNiPolyAAm in LP, at a load of 98 N, the friction coefficient with the introduction of 0.025% FeCoPolyAAm decreases by 20%, and the addition of 0.05% additive reduces the friction coefficient by almost half. The addition of 0.1% FeCoPolyAAm, on the contrary, contributes to an increase in the friction coefficient (Figure 8).

Due to the presence of corrosion fragments on the balls, heterogeneous components can flake off, which increases wear losses. When the corrosion layer is destroyed, heterogeneous components form a loose structure, which during sliding, can move along with friction pairs, acting as an abrasive, and lead to an increase in the friction coefficient and wear losses.

**Figure 9:**
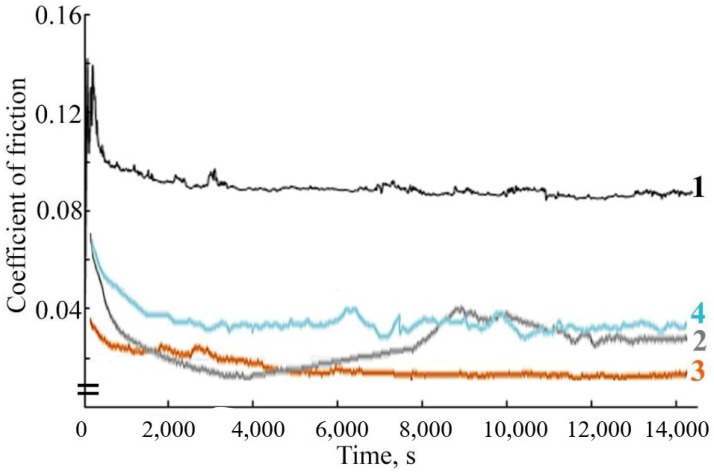
Change in friction coefficient over time when using the lubricant: 1—LP, 2—LP + 0.025% FeNiPolyAAm, 3—LP + 0.05% FeNiPolyAAm, 4—LP + 0.1% FeNiPolyAAm.

The study of the antifriction properties of the lubricant composition during the friction of a steel-steel pair with the addition of FeNiPolyAAm indicates a change in the friction coefficient over time compared with the friction coefficient during frictional interaction in pure LP. The tribological properties of LP improve with the addition of an additive at any concentration. However, it should be noted that the use of FeNiPolyAAm at a concentration of 0.05% reduces the friction coefficient to a greater extent (Figure 9).

### 3.3. Preparation of FeCo/C-N and FeNi/C-N Nanocomposites

**Figure 10:**
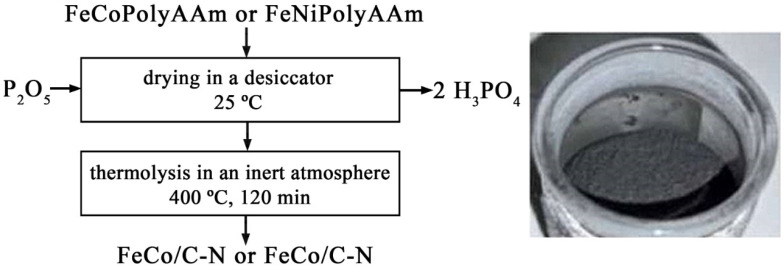
FeCo/C-N and FeNi/C-N nanocomposite synthesis scheme and powder photograph.

Thermolysis of FeCoPolyAAm and FeNiPolyAAm in an inert atmosphere leads to the preparation of FeCo/C-N and FeNi/C-N nanocomposites (Figure 10).

**Figure 11:**
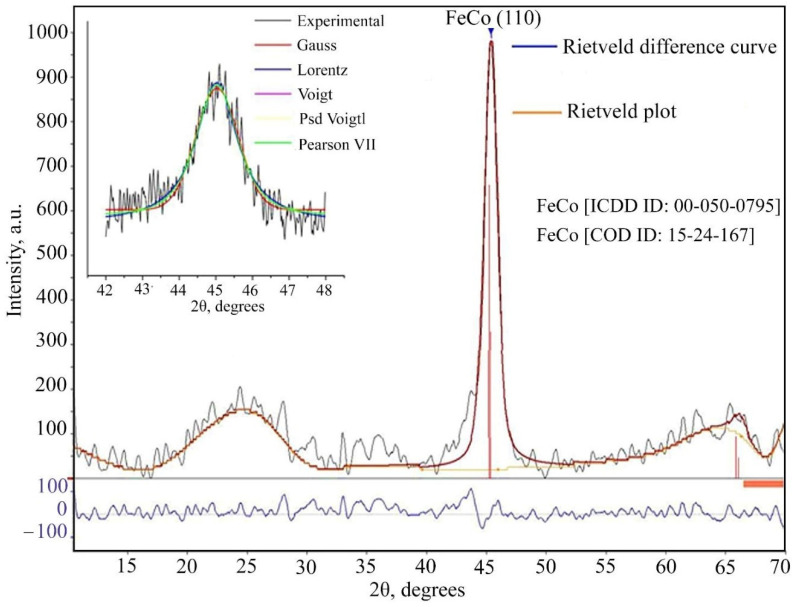
XRD pattern of the FeCo/C-N nanocomposite synthesized at 673 K.

To identify the phase of the obtained products, the exact position of the intensity peak is determined using various models based on the Gaussian, Lorentzian, Voigt, pseudo-Voigt, and Pearson functions. The analysis of the diffraction pattern was carried out by the Rietveld method for two main parameters of the initial spectrum 𝜒^2^, r. By comparing the difference curves for various functions, it was found that the Voight function describes the obtained diffraction pattern as accurately as possible (Figure 11). XRD study of the FeCo/C-N nanocomposite microstructure showed that the diffraction peak at 2*θ* = 45.125° (110) corresponds to the standard diffraction pattern of face-centered cubic FeCo (CODE ID: 15-24-167; ICDD 00-050-0795) (Figure 11). The detected broad diffraction peaks (halos) at 2*θ* = 23°, 35°, and 63° can be attributed to the carbonized polymer shell. According to the calculation of the crystallite size using the Scherrer equation, the average particle diameter is 10 nm.

**Figure 12:**
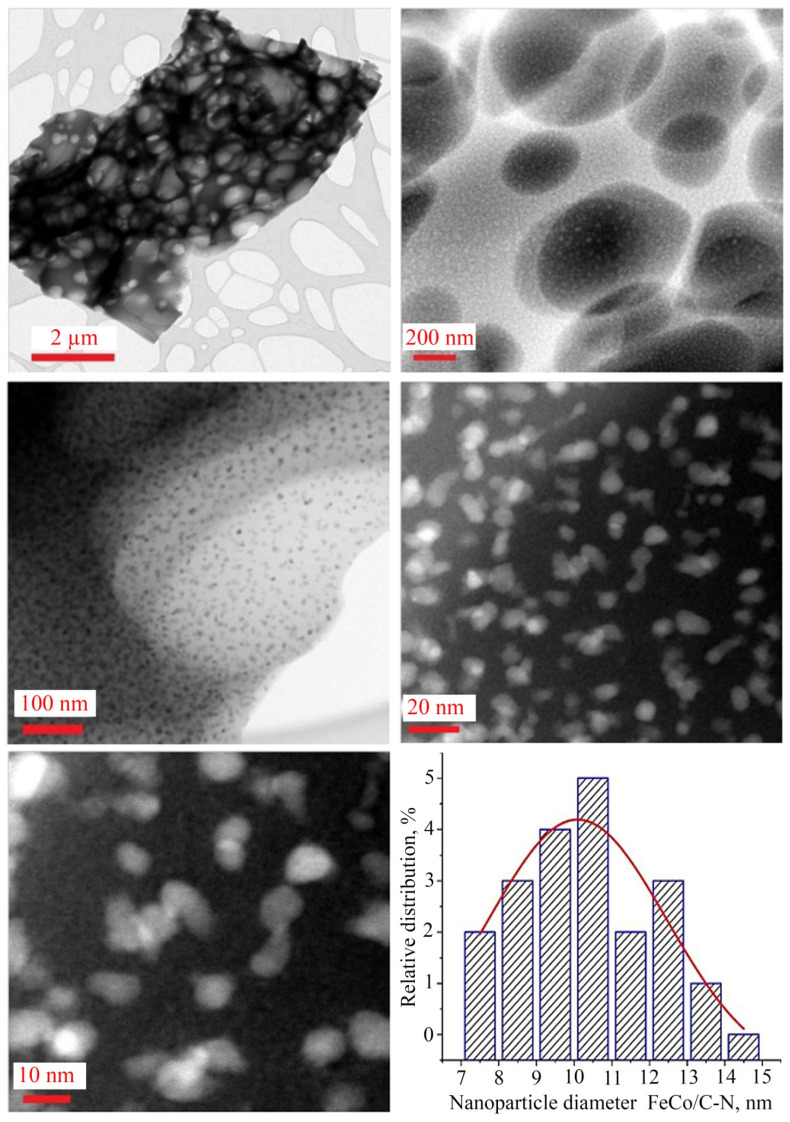
TEM images of NPs and size distribution histogram.

According to TEM data of the nanocomposites, a nitrogen-doped carbonized matrix is formed, which is confirmed by elemental analysis data (Figure 12) with encapsulated spherical FeCo particles with an average size of 10 nm. The carbon shell of the matrix has dimensions from 50 to 400 nm.

**Figure 13:**
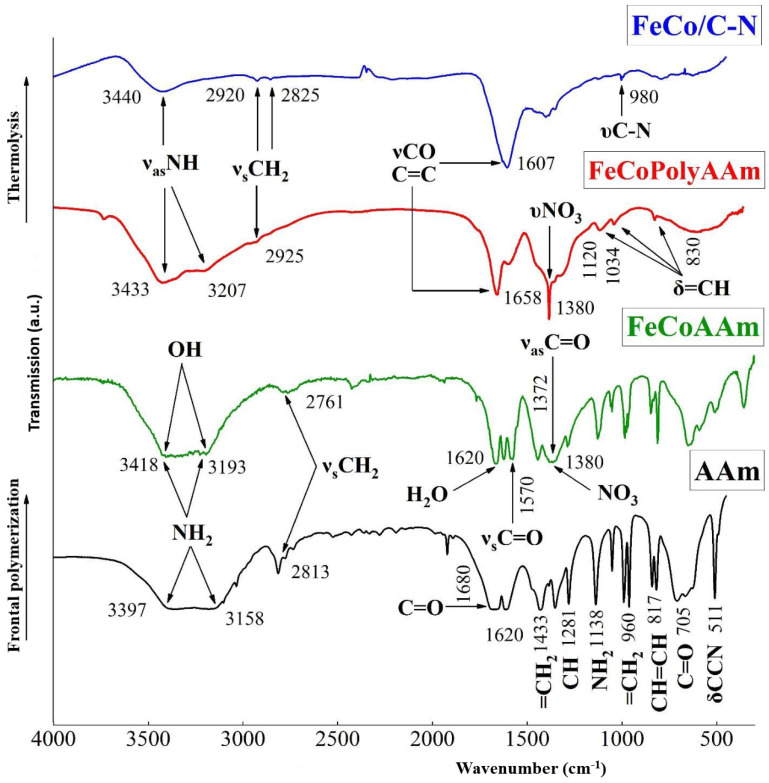
IR spectra of samples: acrylamide AAm, acrylamide complex FeCoAAm, polyacrylamide complex FeCoPolyAAm and FeCo/C-N nanocomposite.

The study of the mechanism of formation of polyacrylamide metal complexes by IR spectroscopy showed a slight shift of the absorption bands (C=O) to the long wavelength region and a decrease in the frequencies of bending vibrations υ(NH) due to complexation with iron(III) and cobalt(II) ions, which indicates coordination of AAm through the oxygen of the carbonyl group. The spectrum of the FeCoPolyAAm polymer complex retains absorption bands in the region of NH stretching vibrations. A significant decrease in the intensity of the absorption bands in the fingerprint region δ(=CH) 980 cm^−1^ confirms the consumption of C=C bonds during polymerization (Figure 13, Table 2). After thermolysis, the initial structure of the polymer complex is destroyed with the preservation of NH stretching vibrations (3440 cm^−1^). Deformation vibrations of NH_2_ (796–850 cm^−1^) and low-frequency vibrations (830 cm^−1^) appear, which indicates the formation of a coordination bond with the metal. All assignments are made on the basis of literature data [39,40]. In the IR spectra of the nanocomposite, there are absorption bands of C=C stretching vibrations characteristic of conjugated systems such as triene and diene fragments in the region of 1606 and 1388 cm^−1^, a wide absorption band in the region of C-N vibrations (3412 cm^−1^), which indicates the formation of a carbonized N-doped shell. All absorption bands have a Gaussian shape, indicating that stretching and bending vibrations occur in a limited space due to a high degree of crosslinking in the system. 

**Figure 14:**
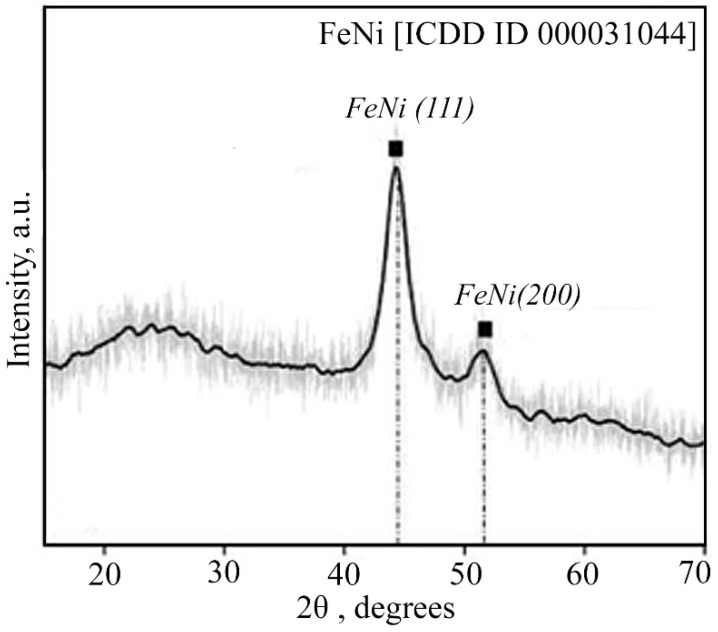
XRD pattern of the FeNi/C-N nanocomposite synthesized at 673 K.

In the resulting FeNi/C-N nanocomposite, the crystalline phase is characterized by two peaks (2*θ* = 44.75° and 52.7°) of nanocrystalline FeNi, identified in the PDF-2-ICDD database, with an average crystallite size of 12 nm (Figure 14). An amorphous halo with a maximum of 23° corresponds to a carbon matrix.

**Figure 15:**
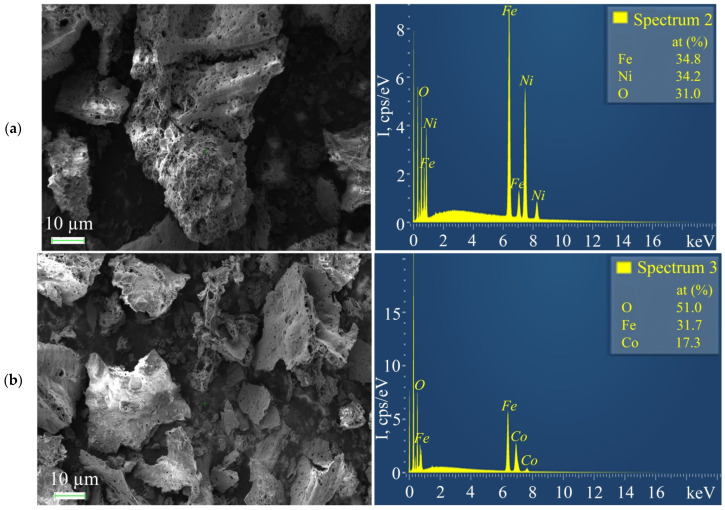
SEM micrographs and EDX analysis of FeNi/C-N (**a**) and FeCo/C-N (**b**) nanocomposites.

The presence of nitrogen in the composition of the composite was established by elemental analysis (Figure 15), which, together with the simplicity of the synthesis of bimetallic polymer complexes by the FP method of their monomeric precursors and the easily controlled thermolysis process, makes it possible to modify the carbon structure with nitrogen.

### 3.4. Tribological Testing of FeCo/C-N and FeNi/C-N Nanocomposites

**Figure 16:**
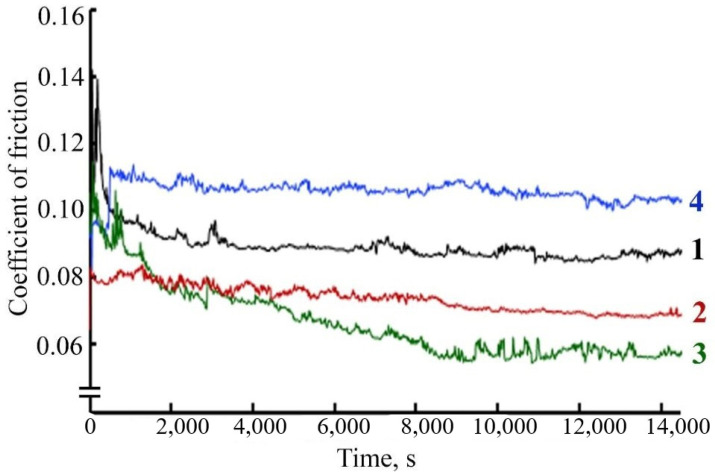
The dependence of the friction coefficient on time during the frictional interaction of a steel-steel friction pair in a lubricating composition: 1—LP, 2—LP + 0.025% FeCo/C-N nanocomposite, 3—LP + 0.05% FeCo/C-N nanocomposite, 4—LP + 0.1% FeCo/C-N nanocomposite.

The study of the antifriction properties of the lubricant composition during friction of a steel-steel pair with the addition of FeCo/C-N or FeNi/C-N nanocomposites indicates a change in the friction coefficient over time compared with the friction coefficient during frictional interaction in pure LP. When a FeCo/C-N nanocomposite with a concentration of 0.05% is added to LP, the friction coefficient values decrease by more than 40% (Figure 16).

**Figure 17:**
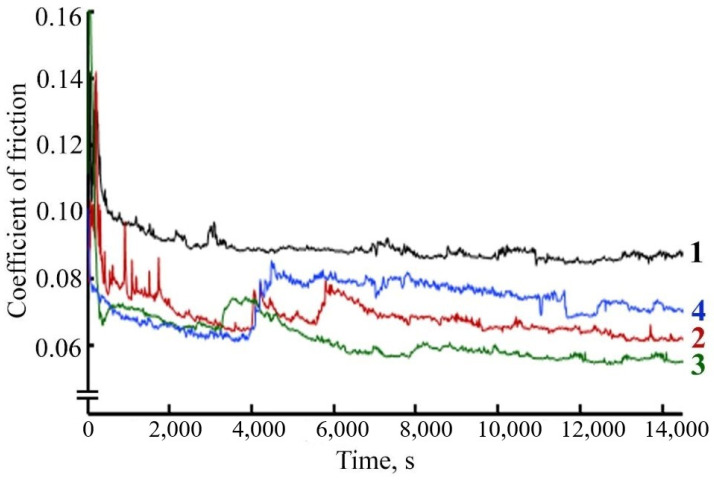
The dependence of the coefficient of friction on time during the frictional interaction of a steel-steel friction pair in a lubricating composition: 1—LP, 2—LP + 0.025% FeNi/C-N nanocomposite, 3—LP + 0.05% FeNi/C-N nanocomposite, 4—LP + 0.1% FeNi/C-N nanocomposite.

A decrease in the values of the friction coefficient is also observed during friction with the addition of FeNi/C-N nanocomposite with a concentration of 0.05% (Figure 17). It should be noted that the addition of FeCo/C-N nanocomposite or FeNi/C-N nanocomposite with a concentration of 0.025% in LP also leads to a decrease in the friction coefficient compared with the value of the friction coefficient during frictional interaction in a pure LP from 0.096 to 0.075 (Figure 16 and Figure 17). An increase in the concentration of FeNi/C-N nanocomposite in LP to 0.1% negatively affects the antifriction properties of the lubricant composition, which leads to an increase in the friction coefficient to 0.11 (Figure 17). An increase in the concentration of FeNi/C-N nanocomposite in LP to 0.1% also contributes to a decrease in the friction coefficient compared with its values during friction in the base oil, however, after 4000 s of frictional interaction, a sharp increase in the values of the friction coefficient is observed by 1.5 times (Figure 17), which may be due to the wear of the friction pair and the abrasive action of a high concentration of NPs in the lubricant composition. The observed fluctuations in the friction coefficient for FeNi/C-N nanoparticles are apparently related to the uneven formation of the tribolayer, since two metal components are involved in this process.

The effectiveness of the synthesized nanoadditives shows that their tribological effect is associated with the properties of NPs as materials with increased surface energy. It follows from the results of tribological tests that NPs contained in a combination of Co and Fe or Ni and Fe (bimetallic nanoalloys FeCo/C-N or FeNi/C-N) are more effective than in the form of a separate additive [40]. Fe NPs in the composition of the composite are important for the formation of a layer that reduces friction and wear and improves adhesion to the bulk material. FeNi/C-N and FeCo/C-N NPs can form a boundary lubricant film on sliding surfaces [41] of steel, penetrating cracks and scratches. The addition of 0.5% FeNi/C-N and FeCo/C-N nanocomposite leads to a decrease in the coefficient of friction, which may be due to the necessary and sufficient number of NPs to form a boundary layer that protects the rubbing surfaces. Increasing the concentration of the additive works like an abrasive.

**Figure 18:**
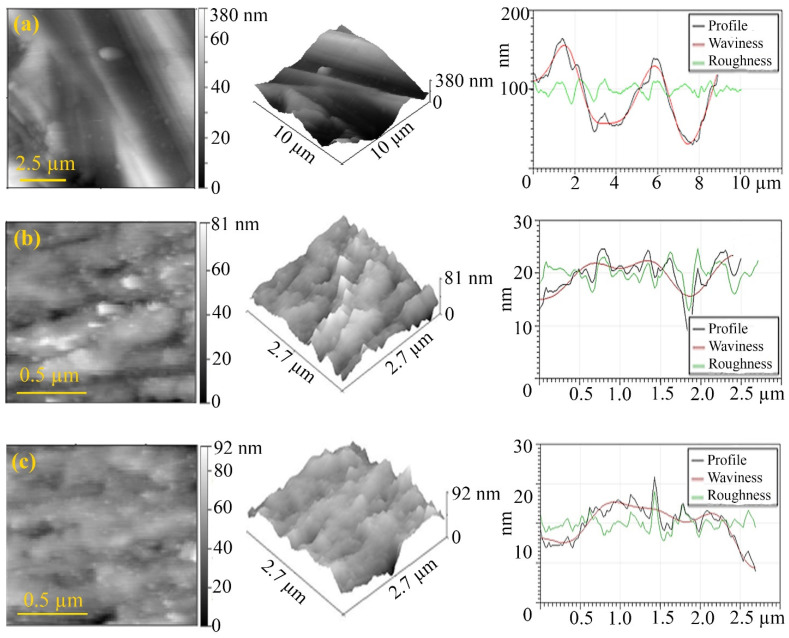
2D, 3D visualization of the AFM image of the friction track: a clean disk (**a**), after friction with the addition of FeCoPAAm (**b**), after friction with the addition of FeCo/C-N nanocomposite (**c**). Additive concentration is 0.05%.

**Figure 19:**
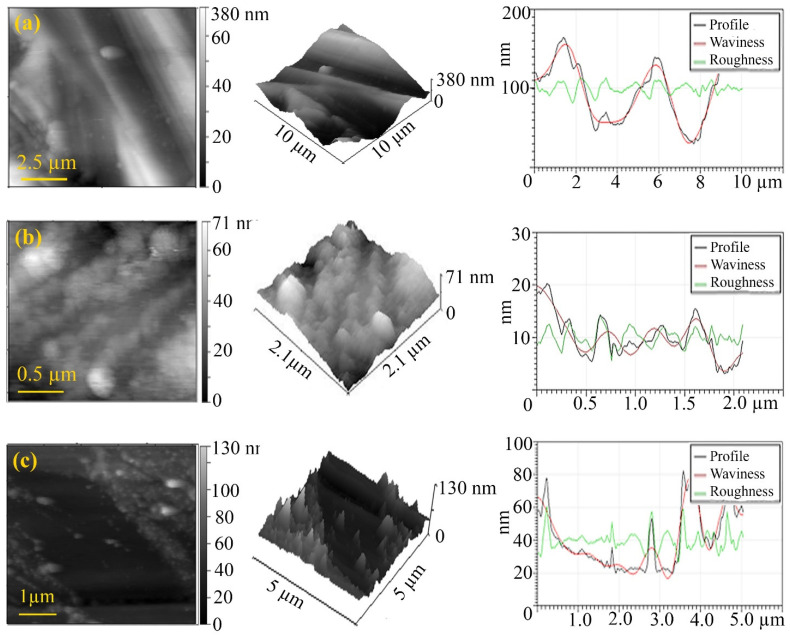
2D-, 3D-visualization of the AFM image of the friction track: a clean disk (**a**), after friction with the addition of FeNiPAAm (**b**), after friction with the addition of FeNi/C-N nanocomposite (**c**). Additive concentration is 0.05%.

An AFM study of the friction track after the frictional interaction of a steel-steel friction pair in the presence of LP with the addition of FeCoPAAm and FeCo/C-N nanocomposite (Figure 18), FeNiPAAm and FeNi/C-N nanocomposite (Figure 19) revealed a decrease in surface roughness compared with the initial surface after frictional interaction with the additives of FeCoPAAm and FeCo/C-N nanocomposite, FeNiPAAm and FeNi/C-N nanocomposite.

The deposition of FeCoPAAm and FeCo/C-N nanocomposite (Figure 18) as well as FeNiPAAm and FeNi/C-N nanocomposite (Figure 19) on the sample surface in the form of an acicular layer. On the friction track, it is smoothed out, which is clearly seen in Figure 19c, while the roughness decreases by almost 2 times. In addition, sealing of the friction surface is detected.

## 4. Conclusions

For the first time, frontal polymerization of acrylamide co-crystallizates of Fe and Ni nitrates was carried out with the formation of the corresponding polyacrylamide complex FeNiPAAm along with FeCoPAAm described earlier. The low-temperature (400 °C) thermolysis of polyacrylamide complexes to obtain a stable bimetallic nanoalloy FeCo/C-N or FeNi/C-N has been demonstrated. In the FeCo/C-N and FeNi/C-N nanocomposites obtained, FeCo and FeNi nanoalloy particles are encapsulated in a carbonized shell of thermolyzed polyacrylamide. The formation of a protective N-doped carbonized shell of FeCo and FeNi nanoparticles makes it possible to increase the manufacturability of the material for oxidation protection since it is not necessary to create a protective atmosphere during the further production of blanks from the material. In addition, the protective matrix forms a phase boundary consisting of polymerized acrylamide, in which the carbon component performs a damping function. In the nanocomposite, the crystalline phase contains FeCo or FeNi nanoparticles and N-doped carbonized polymer shell. The average size of crystallites of FeCo and FeNi nanoparticles is 10 nm and 12 nm, respectively. The carbon shell of the matrix has dimensions from 50 to 400 nm. These results mean that the thermolysis technique via frontal polymerization can encapsulate bimetallic alloy nanoparticles in a C-N layer with suitable composition properties. The resulting nanocomposites were tested as antifriction and antiwear additives in lubricating oils.

## Figures and Tables

**Table 1:** Examples of antifriction materials based on bimetallic nanocomposites.

Number	Composite	Size, nm	Friction Pair	Coefficient of Friction	Ref.
1	Fe-Ni	10	Fe-Ni-silicon wafer	0.220	[17]
2	W/Cu	Bimodal distribution 20 and 700, spherical shape	W/Cu-сталь	0.500	[18]
3	Ni-Fe/Al_2_O_3_	Antifriction coating	Ni-Fe/Al_2_O_3_-steel	0.350	[19]
4	MoO_3_/Bi_2_O_3_	5–65	MoO_3_/Bi_2_O_3_-ZrO_2_	0.220	[20]
5	CoCrNi-Ti	Coating	CoCrNi-Ti-steel	0.360	[21]
6	Fe_3_O_4_/MoS_2_	about 50–200	steel-steel	0.013	[22]
7	ZB/MoS_2_	40–120	steel-steel	0.105	[23]
8	BP/TiO_2_	about 10	steel-steel	0.220	[24]
9	calcium borate/cellulose acetate-laurate nanocomposite	from 10 to 15 μm	steel-steel	0.100–0.150	[25]
10	Al_2_O_3_/SiO_2_	about 70	steel-steel	0.028–0.034	[26]
11	TiO_2_/Al_2_O_3_	about 80	steel-steel	0.040	[27]
12	ZB/MoS_2_	70–100	steel-steel	0.090	[28]
13	Al_2_O_3_/TiO_2_	75	steel-steel	0.050–0.070	[29]
14	ATP-CoNi(C)	70	steel-steel	0.320	[30]

**Table 2:** Characteristic absorption bands (cm^−1^) in IR spectra of acrylamide complexes of metal nitrates and their products of thermolysis.

Compounds	ν_as_NH	ν_s_NH	νH_2_O, δH_2_O	νCO	δNH	δCH_2_	νCH	δ-CH=CH_2_	νNO_3_
AAm	3397	3150	-	1680	1620	1433(νC-N)	1281	960, 817	-
FeCoAAm	3320	3193	3418, 1620	1660, 1372	1570	1440(νC-N), 1430	1285	980	1385
FeCoPAAm	3290	3207	3443	1658, 1372	1580	1440(νC-N)	1120	1034, 830 w	1380
FeNiAAm	3290	3195	3400, 1600	1665	1580 1590	1443	1280	985	1385
FeNiPAAm	3350	3180	3425, 1613	1670, 1372	1555 1580	1280	1285	1034, 830 w	1380
FeCo/C-N	3420	3340	3440, 1600	1607	1595	1430, 980	1280	-	-
FeNi/C-N	3358	3192	3440, 1600	1600	1580	1412, 980	1282	-	-

## Data Availability

The data presented in this study are available on request from the corresponding author.
